# Direct Liver Invasion from a Gastric Adenocarcinoma as an Initial Presentation of Extranodal Tumor Spread

**DOI:** 10.1155/2012/651232

**Published:** 2012-06-17

**Authors:** Mitanshu Shah, Apsara Prasad, Dhyan Rajan, Christopher B. Tan, Mansi Shah, Pooja Raghavan, Paul Mustacchia

**Affiliations:** ^1^Department of Internal Medicine, Nassau University Medical Center, East Meadow, NY 11554, USA; ^2^Department of Gastroenterology, Nassau University Medical Center, East Meadow, NY 11554, USA; ^3^Department of Medicine, New York College of Osteopathic Medicine, Old Westbury, NY 11568, USA; ^4^Department of Internal Medicine, Mount Carmel Health, Columbus, OH 43222, USA

## Abstract

Gastric cancer often carries a poor prognosis, with an estimated 740,000 deaths from the malignancy occurring yearly worldwide (Dicken et al., 2005). The mortality of disease is largely dependent on the extent of tumor spread, as gastric cancer has a predilection to metastasize to other visceral secondaries via hematogenous and lymphatic dissemination. Direct invasion of a gastric adenocarcinoma to adjacent organs secondary to gastric wall perforation does occur; however, it is often present in the setting of advanced disease. Rarely does direct tumor invasion to adjacent organs from a gastric adenocarcinoma present as the initial manifestation of extranodal tumor spread. We present a case of a 40-year-old male with direct tumor extension to the liver as an initial presentation of extranodal tumor spread from a gastric adenocarcinoma. Clinicians should be aware of such an occurrence, as treatment modalities in direct liver extension from a gastric adenocarcinoma vary and may be directed towards palliation rather than curative intent.

## 1. Introduction

Gastric cancer is the second most common malignancy worldwide [1]. Although less prevalent in North America; gastric cancer still accounts for nearly 10, 500 deaths per year [1]. The median survival time after diagnosis is directly proportional to the extent of extranodal tumor spread. Direct invasion of a gastric adenocarcinoma to adjacent organs via perforation of the gastric wall is infrequent and occurs in the setting of widely disseminated disease [2]. Direct tumor extension to the liver at initial presentation of a gastric adenocarcinoma is rare and harbors an extremely poor prognosis [2, 3]. We present a case of a 40-year old male with direct tumor extension to the liver as an initial presentation of extranodal tumor spread from a gastric adenocarcinoma. 

## 2. Case Presentation

A 40-year-old Polish male with no significant medical history presented to the emergency room with complaints of dysphagia and weight loss for nearly 3 months. The patient began noticing difficulty in swallowing liquids, eventually progressing to dysphagia for both liquids and solids over the last several weeks. He described the dysphagia as difficulty passing a food bolus through his lower esophagus and denied any difficulty initiating a swallow. The patient also noticed an unintentional weight loss of nearly 30 pounds over the past 3 months. He denied any nausea, vomiting, abdominal pain, melena, hematemesis, fevers, or chills. The patient denied the use of any medications, illicit drugs, alcohol, or tobacco. Family history was noncontributory, including the absence of any malignancy, gastrointestinal, or neurologic disorders.

Physical examination was remarkable for pallor and mild midepigastric tenderness without guarding or rigidity. Digital rectal examination did not reveal any mass lesions or evidence of gross bleeding. Laboratory evaluation was significant for hemoglobin of 8.9 gm/dL with hematocrit of 27%. Liver transaminases were within normal range; however, there was an increased alkaline phosphatase noted to be 205 U/L. 

**Figure 1:**
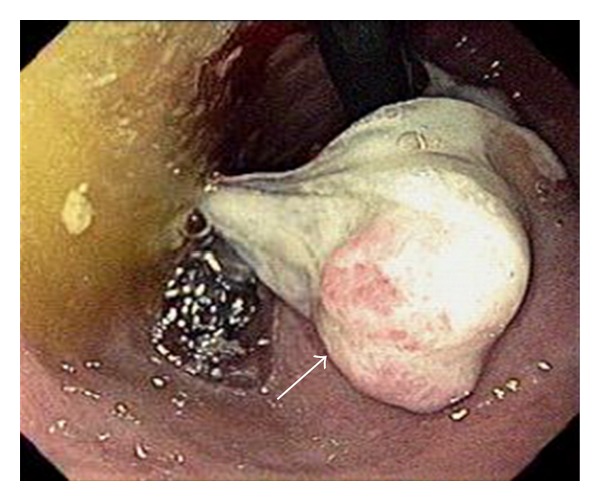
A large, fungating gastric cardia mass (arrow) suspicious for neoplasm seen on esophagogastroduodenoscopy (EGD).

**Figure 2:**
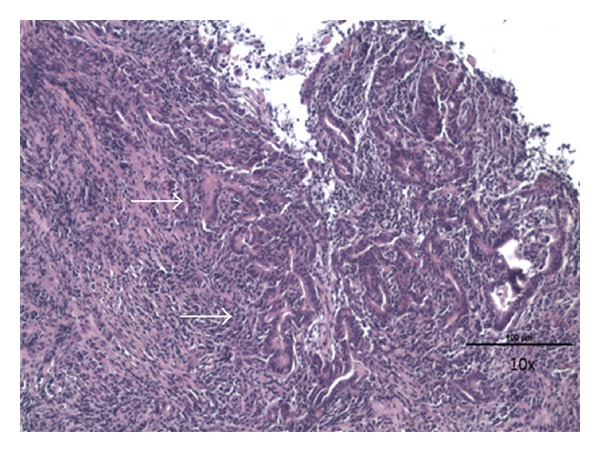
Histopathology of the large gastric mass. Note the irregular gland formation (arrows) highly suggestive of a diffuse-type gastric adenocarcinoma.

**Figure 3:**
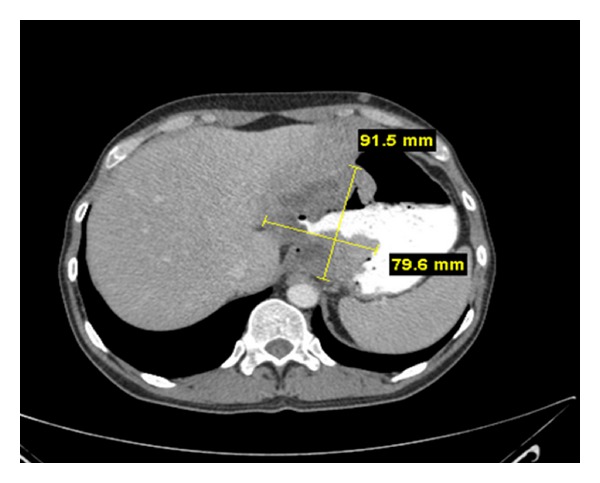
A 9.15 cm × 7.96 cm soft tissue mass within the cardia of the stomach involving the gastroesophageal junction and along the lesser curvature. Note the localized invasion of the tumor into the liver parenchyma.

The patient was admitted to the medical ward where he underwent an esophagogastroduodenoscopy (EGD). EGD revealed a large, ulcerated, fungating gastric cardia mass with overlying exudates suspicious for neoplasm (Figure 1). Several biopsies were obtained which confirmed the presence of a moderately differentiated gastric adenocarcinoma (Figure 2). Computed tomography (CT) of the head, thorax, abdomen, and pelvis was remarkable for a 9.15 cm × 7.96 cm soft tissue mass within the cardia of the stomach involving the gastroesophageal junction and along the lesser curvature. Also noted was extension of the mass into the liver parenchyma suggestive of local tumor invasion (Figure 3). There was no evidence of invasion to any other organs or distant metastases.

Given the size and extension of the gastric mass, the patient was deemed a poor surgical candidate. After an oncology evaluation, patient underwent chemoradiation therapy with paclitaxel and carboplatin. The patient was discharged and continued to follow with the oncology service for further chemoradiation therapy. 

The patient returned to the medical emergency room 2 months later after the abrupt onset of large volume hematemesis. Emergent EGD revealed the invading gastric mass was unchanged from previous endoscopic evaluation 2 months prior. Also observed was the presence of blood clots in the fundus, with no evidence of active bleeding. Repeat CT of the abdomen was suggestive of increasing tumor invasion into the liver parenchyma compared to previous imaging done prior to chemoradiation therapy. The patient refused further diagnostic and therapeutic measures. His hospitalization was complicated by repeated massive hematemesis and multiple organ failure leading to his eventual death on hospital day 6.

## 3. Discussion

Gastric cancer is the second most common cancer worldwide, with the incidence varying with geographical location [1]. Gastric cancer is also currently the second most common type of cancer-related deaths worldwide with the highest incidence in Japan, Eastern Asia, South America, and Eastern Europe, respectively [1]. In North America, gastric cancer is relatively infrequent, yet significantly contributes to the burden of cancer-related deaths [2–5]. In 2010, the American Cancer Society (ACS) reported approximately 21,000 new cases of gastric cancer, with an estimated 10,500 mortalities. As with most malignancies, the severity of disease depends on the stage in which the tumor is first discovered along with the presence of extranodal tumor spread [2]. 

The most common sites of visceral secondaries from a primary gastric adenocarcinoma include the lung and liver with metastases to the brain and bony structures occurring less frequently [1]. Tumor spread to such organs most often involves hematogenous spread and dissemination via the lymphatic system [1–3]. Direct extension of a gastric adenocarcinoma into adjacent structures including the omenta, diaphragm, transverse colon, and duodenum has been reported; however, usually occur it is in the setting of advanced disease and the presence of other visceral secondaries [3]. Direct tumor extension into liver parenchyma from a gastric adenocarcinoma is infrequent and is rarely the initial manifestation of extra nodal tumor spread [2, 3]. 

The classification of gastric adenocarcinoma has proven to be essential in the evaluation of prognoses in patients with this malignancy. According to the Lauren classification, gastric adenocarcinomas may be differentiated into two subgroups; intestinal type and diffuse type [6]. Gastric adenocarcinoma of diffuse type has unorganized tumor cells diffusely infiltrating the stroma of the stomach. These tumor cells often demonstrate deep infiltration of the stomach wall with modest gland formation as seen in our patient. If infiltration of the serosal layer by tumor cells occurs, this may lead to invasion of the tumor to adjacent organs [2, 3]. 

Direct tumor invasion to liver parenchyma from a gastric adenocarcinoma is infrequent and occurs in the setting of advanced disease [2, 3]. In a study by Korenaga et al., 207 patients with direct tumor invasion to adjacent organs secondary to a gastric adenocarcinoma were reviewed. Direct invasion to the liver was found to occur in less than 7 percent of patients with invasion to the pancreas, mesocolon, and peritoneum occurring more frequently [3]. Of the patients noted to have direct extension to the liver, all had evidence of extra nodal tumor spread. Although direct tumor invasion to the liver from a gastric adenocarcinoma often heralds advanced disease, a multitude of therapeutic options have been considered in this patient population. 

Surgical interventions have been attempted in patients with gastric adenocarcinoma with direct liver invasion. In a study by Kunisaki et al., 10 patients with direct liver invasion from a gastric adenocarcinoma were evaluated with 3 patients undergoing curative combined resection of the gastric and liver tumor. Of these 3 patients, median survival time was only 13 months after the procedure with the median survival time without curative surgical intervention being 11 months [7]. The therapeutic benefit of curative surgery may yield low median survival time postoperatively, as it is extremely difficult to resect the invading tumor completely [7]. Given the potentially fatal intraoperative risks and postoperative morbidities associated with curative resection, clinicians should be cognizant of the minimal prolongation of median survival time after this procedure. 

Chemotherapeutic options are available to patients with gastric adenocarcinoma and direct invasion to adjacent organs; however, results are not promising. Randomized trials on combined chemotherapy agents, such as, cisplatin along with bleomycin or etoposide have demonstrated statistically significant improved survival rates over single chemotherapy agents [8, 9]. Clinically combined therapy however, has shown to only prolong median survival time survival 1 month [8–10]. Newer trials appear to favor a three-drug combination with a constant infusion of fluorouracil with cisplatin and an anthracycline; however, the mortality benefit of this regimen appears unclear [10].

Although surgical intervention and chemotherapeutic options appear to have only a nominal benefit on median survival times, they often aid in palliation of symptoms. Noncurative surgical resection may alleviate symptoms of abdominal pain, dysphagia, nausea, and vomiting. Palliative chemotherapy can result in tumor shrinkage, control of neoplastic proliferation and altering tumor biology and metabolic activity; thus systemic and local symptom alleviation may be achieved [11, 12]. Given the high mortality of gastric adenocarcinoma with direct liver extension despite potentially curative interventions, palliative measures may be considered as initial therapy in patients with this aggressive tumor spread. 

## 4. Conclusion

Patients with a gastric adenocarcinoma and direct tumor extension to the liver have a dismal prognosis with 5-year survival rates thought to be less than 15 percent [2, 3]. Although direct extension to the liver from a gastric adenocarcinoma often occurs in the setting of advanced extranodal tumor spread, clinicians should be aware that such a presentation may occur at initial diagnosis of a gastric adenocarcinoma. Preliminary treatment options in such patients should be directed towards palliation, rather than curative intent, as consequences of such tumor extension prove to be almost universally fatal. 

## References

[1] Dicken BJ, Bigam DL, Cass C, Mackey JR, Joy AA, Hamilton SM (2005). Gastric adenocarcinoma: review and considerations for future directions. *Annals of Surgery*.

[2] Roviello F, Rossi S, Marrelli D (2006). Perforated gastric carcinoma: a report of 10 cases and review of the literature. *World Journal of Surgical Oncology*.

[3] Korenaga D, Okamura T, Baba H, Saito A, Sugimachi K (1988). Results of resection of gastric cancer extending to adjacent organs. *British Journal of Surgery*.

[4] Langell JT, Mulvihill SJ (2008). Gastrointestinal perforation and the acute Abdomen. *Medical Clinics of North America*.

[5] Koizumi W, Narahara H, Hara T (2008). S-1 plus cisplatin versus S-1 alone for first-line treatment of advanced gastric cancer (SPIRITS trial): a phase III trial. *The Lancet Oncology*.

[6] Lauren P (1991). Histogenesis of intestinal and diffuse types of gastric carcinoma. *Scandinavian Journal of Gastroenterology*.

[7] Kunisaki C, Akiyama H, Nomura M (2006). Surgical outcomes in patients with T4 gastric carcinoma. *Journal of the American College of Surgeons*.

[8] Ohtsu A, Shimada Y, Shirao K (2003). Randomized phase III trial of fluorouracil alone versus fluorouracil plus cisplatin versus uracil and tegafur plus mitomycin in patients with unresectable, advanced gastric cancer: the Japan Clinical Oncology Group Study (JCOG9205). *Journal of Clinical Oncology*.

[9] Wagner AD, Grothe W, Haerting J, Kleber G, Grothey A, Fleig WE (2006). Chemotherapy in advanced gastric cancer: a systematic review and meta-analysis based on aggregate data. *Journal of Clinical Oncology*.

[10] Yagi Y, Seshimo A, Kameoka S (2000). Prognostic factors in stage IV gastric cancer: univariate and multivariate analyses. *Gastric Cancer*.

[11] Kim A, Fall P, Wang D (2005). Palliative care: optimizing quality of life. *Journal of the American Osteopathic Association*.

[12] Coia LR, Hanks GE, Martz K, Steinfeld A, Diamond JJ, Kramer S (1988). Practice patterns of palliative care for the United States 1984-1985. *International Journal of Radiation Oncology Biology Physics*.

